# Circadian Regulation and Pain: A Systematic Review of the Association Between Rest–Activity Rhythm and Pain-Related Outcomes

**DOI:** 10.3390/clockssleep8020032

**Published:** 2026-05-28

**Authors:** Aline Van Stallen, Manon De deyne, Céline Labie, Liesbet De Baets

**Affiliations:** 1 Musculoskeletal Rehabilitation Research Group, Department of Rehabilitation Sciences, Faculty of Movement and Rehabilitation Sciences, KU Leuven, 3000 Leuven, Belgium; aline.vanstallen@kuleuven.be (A.V.S.); manon.de.deyne@vub.be (M.D.d.); celine.labie@vub.be (C.L.); 2 Flanders Research Foundation—FWO, 1000 Brussels, Belgium; 3 Pain in Motion Research Group (PAIN), Department of Physiotherapy, Human Physiology and Anatomy, Faculty of Physical Education & Physiotherapy, Vrije Universiteit Brussel, 1050 Ixelles, Belgium; 4 Department of Physical and Rehabilitation Medicine, University Hospitals Leuven, 3000 Leuven, Belgium; 5 Leuven Algologic Center, University Hospitals Leuven, 3000 Leuven, Belgium

**Keywords:** rest–activity rhythm, circadian rhythm, actigraphy, pain

## Abstract

The rest–activity rhythm (RAR) is a key marker of circadian regulation and is commonly assessed using actigraphy. Emerging evidence suggests that characteristics of RAR, such as amplitude, stability, and regularity, may be associated with pain-related outcomes. However, no systematic review has yet synthesized this evidence across populations and pain conditions. This systematic review aimed to provide an overview of current approaches to measuring and defining RAR and to examine its associations with pain outcomes in both healthy individuals and clinical populations experiencing acute or chronic pain. A systematic search of PubMed, Web of Science, Scopus, and Embase was conducted, with the final search completed on 20 May 2025. Observational studies reporting associations between at least one RAR characteristic and a pain outcome were eligible. Article selection and risk-of-bias assessment using the ROBINS-E tool were performed independently by two reviewers, and findings were synthesized narratively. Seven cross-sectional studies were included, employing diverse analytic methods such as cosinor and non-parametric analyses. Overall, the findings were heterogeneous, suggesting that associations between RAR and pain vary according to the RAR metric used, the analytical approach, and the population studied. Nevertheless, the evidence generally indicates that more robust and well-consolidated circadian rhythms are associated with lower pain, whereas regularity and timing appear to play more context-dependent roles, highlighting the potential relevance of RAR metrics as modifiable targets and the need for standardized measurement approaches.

## 1. Introduction

Pain is a universal human experience and a key reason for seeking medical care [1,2]. Chronic pain in particular significantly impairs quality of life, daily functioning, and psychological well-being [2,3,4]. Due to the high prevalence of chronic pain and its substantial socioeconomic burden, it represents a major public health concern [5,6].

Pain is defined as an unpleasant sensory and emotional experience associated with actual or potential tissue damage [7] and is shaped by biological, psychological, and social factors [7]. Pain is also dynamic, with its perceived intensity and unpleasantness fluctuating within a day in most people with chronic pain [8,9]. For example, postoperative pain, fibromyalgia, trigeminal neuralgia, and migraines are described to peak in the morning [9], while tension-type headaches are more frequent in the afternoon and temporomandibular joint pain and neuropathic pain typically worsen in the evening [9]. Such temporal within-day fluctuations matter clinically because they might shape within-day pain-related movement behaviour or inform ideal timing of medication [8,9].

In constant-routine experiments that minimize behavioural and environmental influences, human pain-modulatory pathways follow a robust, endogenous 24 h rhythm [8]. This rhythm is generated by the circadian timing system, an intrinsic network of biological clocks that coordinates rhythms across nearly all physiological systems [10]. The circadian timing system comprises a central pacemaker in the suprachiasmatic nucleus, numerous peripheral clocks in organs and cell types, and input–output pathways that keep these clocks synchronized [10], with the molecular basis of each cellular clock being a set of clock-gene feedback loops that oscillate every ~24 h [10]. Yet, circadian rhythms are also shaped by environmental factors such as light–dark exposure, meal timing and sleep–wake scheduling [11,12,13], which provide input to the circadian clock and thereby influence biological rhythms [11,12,13]. With regard to sleep–wake scheduling, the rest–activity rhythm (RAR) is a core behavioural factor of the circadian timing system [14,15].

Modern wearable technology enables non-invasive monitoring of the RAR in real-world settings [16]. Actigraphy, in particular, uses compact accelerometer-based devices worn continuously over multiple days to estimate sleep and (variations in) daytime activity [16,17,18]. Over time, several metrics have been proposed to quantify RAR from accelerometry data [19,20]. These metrics are typically derived using either parametric or non-parametric approaches. Parametric methods assume that the rhythm follows a mathematical model, typically a sinusoidal curve [19]. Parametric metrics derived from cosinor analysis include Mesor, which represents the average activity level within a 24 h cycle; Amplitude, which reflects how pronounced the contrast is between daytime activity and nighttime rest; and Acrophase, which indicates the timing of peak activity within the cycle [19,20,21]. The goodness-of-fit of the cosinor model is expressed by R^2^, while the Circadian Quotient represents the ratio of Amplitude to Mesor, indicating the relative strength of the rhythm [20]. Non-parametric methods make no assumptions about the shape of the rhythm [19]. Non-parametric metrics include Interdaily Stability, which captures how consistent the rhythm is across multiple days, while Intradaily Variability reflects how fragmented the rhythm is within a day. Relative Amplitude, on the other hand, represents the difference between the most active and least active periods, and the Dichotomy Index (I < O) compares activity levels during in-bed versus out-of-bed periods. Finally, R24 measures how similar the activity pattern is from one day to the next, based on a 24 h autocorrelation [19,20,21].

When behavioural rest–activity patterns become misaligned with the endogenous circadian timing system—shift work being an extreme example—disrupted RARs arise [22]. Such misalignment is expressed in accelerometry-derived rhythms as a weakened day–night contrast, increased intradaily fragmentation, and greater day-to-day variability [22]. Research suggests that disrupted RARs are associated with poorer health outcomes, such as higher risks of incident cancer [23]; cardiovascular, infectious, respiratory, and digestive diseases [22]; dementia and cognitive impairment [24,25]; depression [26]; and all-cause and cause-specific mortality [23]. The association with pain-related outcomes is less frequently reported in the literature. It has been hypothesized that circadian timing disruption can exacerbate pain, while pain itself may further disturb circadian timing, suggesting a potentially bidirectional relationship [8]. However, to date, there is no integrated synthesis of how RAR—as a core behavioural factor of the circadian timing system—relates to pain-related outcomes. This review addresses that gap by systematically synthesizing evidence on how RAR characteristics relate to pain outcomes (pain intensity, sensitivity, duration, frequency, and interference) across diverse populations. We also map and compare actigraphy-based methods used to quantify RAR in pain populations, including preprocessing choices and commonly used metrics. Together, these aims provide a coherent framework for interpreting RAR characteristics as markers of circadian health in pain research and highlight methodological considerations relevant for future studies and clinical translation.

## 2. Results

### 2.1. Study Selection Process

Our literature search resulted in 9794 records in total. After removing duplicates, 5680 unique studies remained and were screened based on title and abstract. Title and abstract screening retrieved 254 records for full-text evaluation.

**Figure 1:**
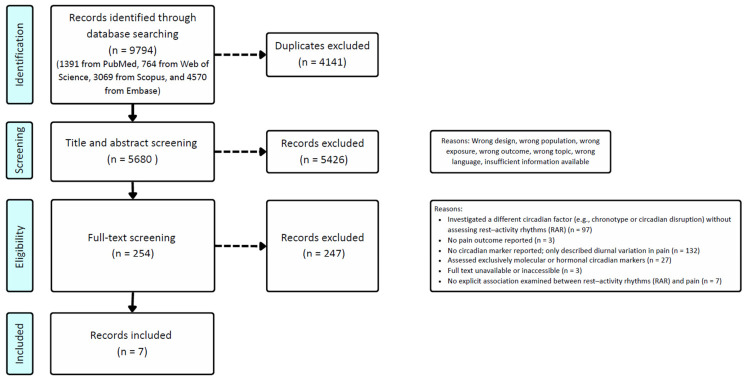
Flowchart for study selection process and reasons for exclusion. Abbreviations: RAR, rest–activity rhythm.

Of the 254 full-text articles assessed, seven studies met all inclusion criteria and were included in the qualitative synthesis. An overview of the study selection process and reasons for exclusion is provided in Figure 1.

### 2.2. Study Characteristics

The seven studies included were published between 2014 and 2025 and originated from diverse geographical regions, including North America and Asia (Canada, the United States, and Taiwan). Four studies [27,28,29,30] adopted a cross-sectional observational design; three [31,32,33] were longitudinal observational studies. Sample sizes ranged from 37 to 292 participants. The study populations were heterogeneous, encompassing individuals with advanced cancer [27,29], those experiencing cancer-related pain [31], patients with fibromyalgia [30], individuals with inflammatory bowel disease [28], patients with temporomandibular disorders [32], and endometrial cancer survivors [33]. Two included studies used data from the same cohort of 68 hospitalized cancer patients with pain, recruited from the oncology ward of a teaching hospital in Taiwan, with Chang & Lin (2014) explicitly stating that their study was part of a larger investigation whose results had previously been published by Ma et al. (2014) [29,31]. Mean participant ages ranged from 37 to 68 years, and the proportion of female participants varied between 47.3% and 100%. Study settings and recruitment strategies were diverse, including community-based, outpatient, and inpatient hospital settings. Detailed inclusion and exclusion criteria and additional study characteristics are summarized in Table 1.

### 2.3. Risk of Bias

**Figure 2:**
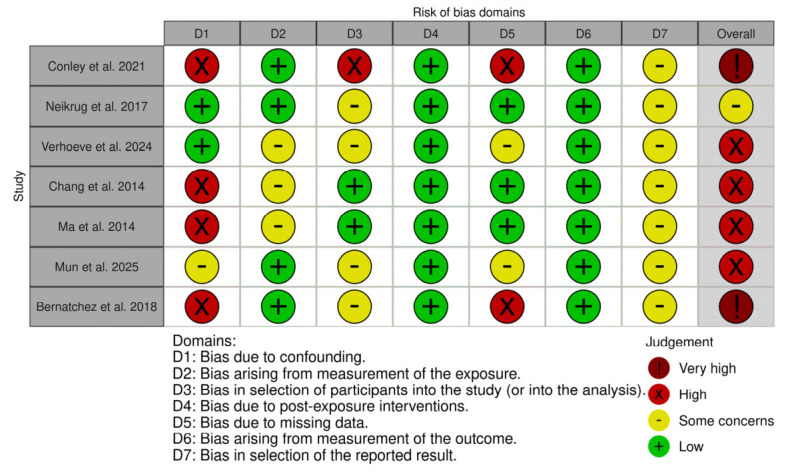
Results of the risk of bias assessment for each included study [27,28,29,30,31,32,33], showing domain-specific ratings and overall judgement.

The resulting κ value (κ = 0.78) indicated a high level of consistency between reviewers’ assessments. The results of the risk of bias assessment for each study are shown in Figure 2 [34], which includes both the domain-specific scores and the overall judgment. One study was rated as having *some concerns* [30], four as *high risk of bias* [29,31,32,33], and two as *very high risk of bias* [27,28].

All studies received a *low risk of bias* rating for the domains *post-exposure interventions* and *outcome measurement*. In contrast, all studies scored *some concerns* for *selective reporting*, mainly because none of them had a predefined analysis plan or study protocol. However, there was no further indication that the reported effect estimates were selectively chosen based on the desirability of the results. Risk of bias related to *confounding*, *exposure measurement*, *participant selection*, and *missing data* varied across studies. In the domain of *confounding*, four studies [27,28,29,31] did not account for relevant confounding factors and were therefore rated as having a *high risk of bias*. One study [32] did control for time-invariant confounders but failed to adjust for important time-varying ones, resulting in a rating of *some concerns*. In the domain of *exposure measurement*, three studies [29,31,33] were rated as having *some concerns* due to a limited actigraphy period (less than the recommended seven days [35]). The greatest bias in *participant selection* came from excluding participants after the exposure window based on factors related to the exposure itself, such as extreme patterns in actigraphy (outliers), non-adherence, or missing data. Finally, in the *missing data* domain, most issues were related to missing values in the exposure data, particularly in the actigraphy recordings.

### 2.4. Assessment of Pain Outcomes

Reported pain-related constructs were pain intensity, interference with daily activities, and daily or 24 h worst pain severity. The specific dimensions and instruments varied considerably across studies. An overview of all pain instrument characteristics can be found in Table 2.

Three studies [29,31,33] employed the Brief Pain Inventory (BPI) or its Chinese adaptation (BPI-C), a widely used self-report questionnaire designed to assess both pain intensity and pain interference with daily functioning [36]. It includes four 0–10 Numeric Rating Scale (NRS) items assessing pain at its “worst in the last 24 h”, “least in the last 24 h”, “average”, and “now”, with a 0 indicating “no pain” and 10 representing “pain as bad as you could imagine” [36]. The remaining seven items measure the extent to which pain interferes with general activity, mood, walking ability, normal work, relations with other people, sleep, and enjoyment of life, again using a 0–10 NRS [36]. For the interference items, 0 represents “does not interfere” and 10 indicates “interferes completely” [36]. Despite using the same instrument, scoring and interpretation differed. Ver Hoeve et al. (2024) computed composite scores for intensity and interference and applied a clinical cut-off (≥5) to classify elevated pain [33]. Ma et al. (2014) also calculated composite scores for intensity and interference but treated them exclusively as continuous variables without categorical thresholds [29]. Chang & Lin (2014) focused solely on pain intensity and categorized scores into mild (1–4), moderate (5–6), and severe (7–10) [31].

Bernatchez et al. (2018) used a single-question pain diary based on the BPI, asking participants to rate their worst pain in the past 24 h on a 0–10 scale (0 = no pain, 10 = worst imaginable) [27]. Mun et al. (2025) assessed daily pain severity in a similar way, using an end-of-day NRS (0 = no pain, 10 = worst imaginable pain) to rate the average level of pain experienced throughout the day [32]. Pain was broadly defined to include any source, ensuring that comorbid pain commonly experienced in people with temporomandibular disorders was captured [32].

In inflammatory bowel disease patients, Conley et al. (2021) measured abdominal pain and other gastrointestinal symptoms over the previous seven days using Patient-Reported Outcomes Measurement Information System (PROMIS) gastrointestinal symptom scales [28]. Finally, in fibromyalgia patients, Neikrug et al. (2017) assessed pain using the pain severity scale from the Multidimensional Pain Inventory (MPI) [30]. This scale assesses how pain interferes with different daily life activities and how severe it was over the last week [30,37].

### 2.5. Assessment and Data Processing Methods of RAR

#### 2.5.1. Measurement Instruments of RAR

RARs were assessed using wrist-worn actigraphy devices. Across studies, devices and protocols varied as follows:

All studies measured RAR using actigraphy, typically worn on the non-dominant wrist. Bernatchez et al. (2018) used the Actiwatch-64^®^ (Philips Respironics, Murrysville, PA, USA) with 30 s epochs, worn continuously for seven days [27]. Chang & Lin (2014) and Ma et al. (2014) used Ambulatory Monitoring Inc. (Ardsley, NY, USA) actigraphs with epochs from one minute, worn for at least three consecutive days [29,31]. Conley et al. (2021) employed the Actiwatch Spectrum Plus (Philips Respironics) with 30 s epochs, worn for ten consecutive days [28]. Mun et al. (2025) used the Actigraph GTX3 (Actigraph LLC, Pensacola, FL, USA) with 1 min epochs, worn continuously for fourteen days [32]. Neikrug et al. (2017) utilized the MicroMini-Motionlogger Actigraph (Ambulatory Monitoring) with 1 min epochs, worn for seven consecutive days [30], while Ver Hoeve et al. (2024) used the Actiwatch-64 (Mini-Mitter, Bend, OR, USA) with 1 min epochs for at least three consecutive days at each time point [33].

All studies supplemented actigraphy with sleep diaries completed by the participants to verify sleep–wake times and off-wrist periods. The devices’ software (e.g., Actiware v6, Action4 software, Action3 software, ActiLife v6 7.3) was used to preprocess data and detect off-wrist intervals.

#### 2.5.2. Metrices of RAR

RAR metrics were derived using both parametric and non-parametric methods [19].

**Figure 3:**
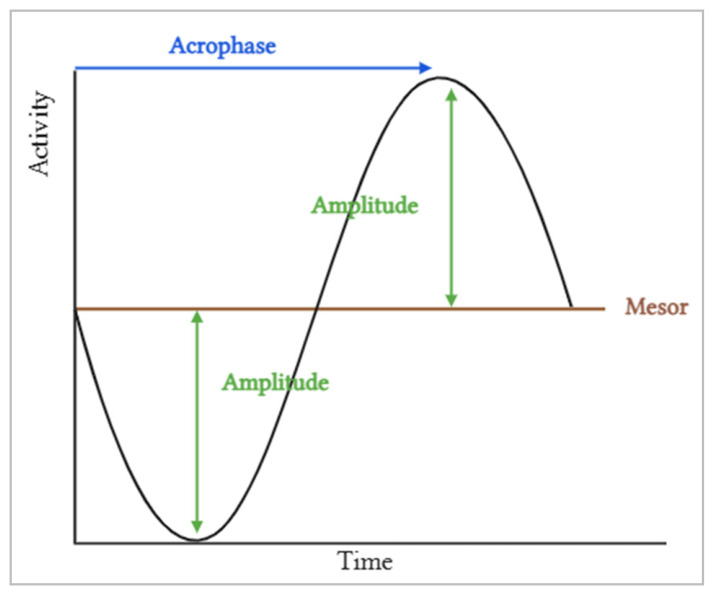
Schematic illustration of a cosinor curve. Mesor represents the rhythm-adjusted mean, Amplitude the maximal deviation from this mean, and Acrophase the timing of the peak.

Parametric measures included Mesor (M), Amplitude (A), Acrophase or Phi (φ), R-squared (R^2^), and Circadian Quotient (CQ), which were reported by Bernatchez et al. (2018), Ver Hoeve et al. (2024), Neikrug et al. (2017), and Conley et al. (2021) [27,28,30,33]. The core cosinor parameters M, A, and φ are illustrated in Figure 3. Non-parametric measures included Interdaily Stability (IS), Intradaily Variability (IV), and Relative Amplitude (RA), as used in Conley et al. (2021) and Mun et al. (2025) [28,32]. The Dichotomy Index (I < O), which compares activity in bed versus out of bed, was employed in Chang & Lin (2014) and Ma et al. (2014) [29,31]. Ma et al. (2014) also calculated the autocorrelation coefficient (R24), which represents the autocorrelation of activity counts at a 24 h lag [29]. An overview of the RAR metrics is provided in Table 3.

#### 2.5.3. Software, Algorithms and Procedures to Analyse RAR

The analysis of RARs in the included studies relied on a combination of data preprocessing, rhythm detection algorithms, and statistical modelling procedures. Although all studies employed wrist-worn actigraphy to capture continuous activity data, the specific analytical methods varied depending on the objectives of each study and the metrics used for characterizing RAR. RAR assessment, data processing methods, and analyses are summarized in Table 4.

Data preprocessing formed in all studies a crucial initial step to ensure the validity of the actigraphy recordings. Raw activity data were downloaded from Actigraph devices using software such as Actiware v6 (Philips Respironics) [27,28,33], Action4 (Ambulatory Monitoring Inc.) [29,30], ActiLife v6 7.3. (Actigraph LLC.) [32], or Action3 [30]. Non-wear periods were first identified automatically using built-in detection algorithms (e.g., the Wear Time Validation function in ActiLife). Subsequently, trained research staff manually verified these periods by comparing them with information from participants’ diaries. Diaries or event markers were consistently used to define lights-off and lights-on periods, nap intervals, and times of device removal.

Following wear-time validation, studies consistently applied either the Cole–Kripke [38] or Sadeh [39] sleep–wake detection algorithms to the actigraphy data. While the algorithms themselves were similar across studies, the operational definitions of sleep onset and wake onset varied. For example, Mun et al. (2025) defined sleep onset as the first sequence of at least five consecutive minutes scored as sleep, and wake onset as the last sequence of at least five minutes of sleep preceding a sustained wake period [32]. In contrast, Bernatchez et al. (2018) relied on sleep diaries and manually scored sleep–wake periods based on participants’ reported bedtimes and rise times [27]. Ver Hoeve et al. (2024) also used sleep logs to document sleep–wake patterns and off-wrist periods, excluding the latter from scoring [33]. Neikrug et al. (2017) also incorporated self-reported electronic sleep logs, using participants’ “Down” (time in bed attempting to sleep) and “Up” (out-of-bed) timestamps to constrain the scoring window; within these boundaries, the Sadeh algorithm was applied to classify each minute as asleep or awake [30]. Conley et al. (2021) used a different approach: sleep–wake periods were scored by a single trained scorer, based on participant-triggered event markers, supported by sleep diaries, and verified using the device’s light sensor [28]. Ma et al. (2014) and Chang & Lin (2014) did not clearly describe how sleep–wake scoring was performed, limiting the comparability of their methods to other studies [29,31].

Once the time series were preprocessed and scored as wake or sleep, rhythm analysis was conducted using two primary methodological frameworks: cosinor modelling (parametric) and non-parametric analysis of activity profiles.

The parametric cosinor model assumes that the circadian rhythm follows a sinusoidal pattern with a 24 h periodicity. This model was applied in the studies of Bernatchez et al. (2018), Conley et al. (2021), Neikrug et al. (2017) and Ver Hoeve et al. (2024), where the actigraphy data from several consecutive days were averaged into a single 24 h profile representing each participant’s overall rhythm [27,28,30,33]. A cosine function was then fitted to this averaged curve to estimate key rhythmic metrics, including the Mesor (M), the Amplitude (A), and the Acrophase (ϕ). From these, additional metrics such as the Circadian Quotient (CQ), R^2^, and R24 were also calculated.

In Bernatchez et al. (2018), model fitting was performed using the extended five-parameter cosinor model in SAS (versions 9.2–9.3) or SPSS (version 24), which allows for more flexibility in capturing asymmetries in activity curves [27]. The R*^2^* statistic was derived to quantify the goodness-of-fit of the model, indicating the proportion of variance in activity explained by the rhythmic component. Higher R^2^ values reflected more robust, regular 24 h activity cycles, while lower values suggested irregular or flattened rhythms.

$$Y\left(t\right)=M+A\cos\left(\frac{2\pi \left(t-\varphi \right)}{T}\right)+\epsilon (t)$$

where *Y*(*t*) is the activity level at time *t*, *M* (Mesor) is the midline estimating statistic of rhythm, *A* represents the Amplitude (the extent of predictable change within the 24 h cycle), *ϕ* is the Acrophase (the timing of the rhythm’s peak), and *τ* is the assumed period, typically fixed at 24 h for circadian analysis.

In contrast, Neikrug et al. (2017) fitted the cosinor model to each individual 24 h day rather than to a single averaged profile [30]. This approach yielded a set of daily parameter estimates (e.g., Amplitude, Mesor, and Acrophase) for each participant. The variability of these metrics across days, expressed as the standard deviation or standard error, was then calculated to quantify intra-individual rhythm stability. In this way, researchers could assess not only the average characteristics of a person’s rest–activity rhythm but also how consistent or variable those rhythms were from day to day.

Non-parametric analyses were used when the data did not follow a regular sinusoidal pattern, which often occurs in individuals with disrupted circadian rhythms. These analyses, as implemented by Chang & Lin (2014), Conley et al. (2021), Ma et al. (2014), and Mun et al. (2025), relied on direct computation of rhythm metrics from the raw activity data without assuming any functional form [28,29,31,32]. The most frequently reported non-parametric variables included Interdaily Stability, Intradaily Variability, Relative Amplitude, R24 and the Dichotomy Index.

**Table 4:** Summary of the assessment, data processing methods, and analyses of rest–activity rhythms (RAR) for each included study.

Study	RAR Metric	Measurement Type	Details Measurement	Epoch Length and Sampling Rate	Recording Duration	Software or Algorithms Used
Bernatchez et al. 2018 [27]	Amplitude, Acrophase, Mesor, Up-Mesor (h), Down-Mesor (h), R-squared	Actigraphy: Actiwatch-64^®^ (Philips Respironics, Andover, MA, USA)	No more information	30 s	24 h per day for 7 consecutive days	Data preprocessing and wear-time validation: Actigraphy data were manually scored within 30 s epochs using sleep diaries. Off-wrist periods were excluded based on diary information and visual inspection. Interrater agreement was excellent (ICC = 86.2–96.3%), and only traces scored by the primary rater were retained. Data were double-entered to ensure accuracy and subsequently aggregated to 60 s epochs by averaging to reduce outliers and smooth time-series data. Analyses: RARs were analysed using the five-parameter extended cosinor model, extracting Amplitude, Acrophase, Mesor, and R^2^. Group differences were assessed with independent-sample t tests and Hedges’ g effect sizes. Analyses included only participants with a rhythmicity coefficient (R^2^) > 5%.
Chang & Lin 2014 [31]	Dichotomy Index I < O Cut-off used: 89.5% (according to the procedure described by Innominato et al. [40])	Actigraphy: Ambulatory Monitoring Inc., New York, NY, USA	No more information	/	Three consecutive days	Data preprocessing and wear-time validation: Actigraphy data were recorded using Ambulatory Monitoring Inc. devices and processed in Action4 software (zero-crossing mode). Daily sleep logs were used to identify bedtimes and wake times, supporting the scoring of rest–activity periods. Analyses: RARs were quantified using the Dichotomy Index (I < O). A median cutoff of 89.5% was used to classify patients into regular (≥89.5%) versus disrupted (≤89.4%) RARs. Analyses focused on daily RARs.
Conley et al. 2021 [28]	Mesor, Amplitude, Acrophase, Circadian Quotient, Interdaily Stability, Intradaily Variability	Actigraphy: Actiwatch Spectrum Plus, Phillips Respironics, Inc.	Worn on non-dominant wrist	30 s	10 consecutive days	Data preprocessing and wear-time validation: Actigraphy data were recorded in 30 s epochs using Actiwatch Spectrum Plus (Phillips Respironics) and scored by a single trained rater. Scoring considered event markers, sleep diaries, and the Actigraph light sensor to define rest periods (lights off to lights on). Data were cleaned and scored using Actiware v6 software following standard procedures. Analyses: RARs were analysed by fitting a cosine curve to 24 h activity data. Extracted metrics included Amplitude, Acrophase, Mesor, and Circadian Quotient. Interdaily Stability and Intradaily Variability were calculated using nonparametric methods. Analyses were conducted in SAS v9.2 using a custom macro.
Ma et al. 2014 [29]	Autocorrelation coefficient at 24 h (R24), Dichotomy Index I < O	Actigraphy: Ambulatory Monitoring Inc., New York, NY, USA	Worn on non-dominant wrist	1 min	At least three consecutive days	Data preprocessing and wear-time validation: Actigraphy data were processed in zero-crossing mode using Action4 software. Patients kept daily sleep logs to record bedtimes and wake times, which supported the scoring of rest–activity periods. Data from complete 3-day recordings were averaged across up and down periods for analysis. Analyses: RARs were assessed using the autocorrelation coefficient at 24 h (R24) and the Dichotomy Index (I < O). For each participant, mean values were calculated from actigraphy data and sleep logs were collected over three consecutive days.
Mun et al. 2025 [32]	Intradaily Variability, Relative Amplitude	Actigraphy: Actigraph GT3x1; ActiGraph LLC, Pensacola, FL, USA	Worn on non-dominant wrist	1 min	24 h per day for 14 consecutive days	Data preprocessing and wear-time validation: Actigraphy data were processed in ActiLife v6.7.3. Off-wrist periods were detected using the Wear Time Validation function. Sleep–wake periods were scored using the Cole–Kripke algorithm, with sleep onset defined as the first ≥5 consecutive min of sleep and sleep offset as the last ≥5 consecutive min of sleep. Scoring was manually verified by trained staff, cross-checked with sleep diaries, and corrections were applied for deviations > 60 min or split nights. Analyses: RARs were analysed using the ActCR R package, based on Axis 1 actigraphy data. Two nonparametric metrics were calculated: Relative Amplitude and Intradaily Variability.
Neikrug et al. 2017 [30]	Amplitude, Acrophase, Mesor, Amplitude Variability, Acrophase Variability, Mesor Variability	Actigraphy: MicroMini-Motionlogger Actigraph (Ambulatory Monitoring, Ardsley, NY, USA)	Worn on non-dominant wrist	32 Hz, 1 min	7 consecutive days	Data preprocessing and wear-time validation: Actigraphy data were downloaded using Action-3 software (Ambulatory Monitoring Inc.) and trimmed to 6 full 24 h periods (noon-to-noon) to remove first-day bias and travel-related activity. Sleep intervals were defined using electronic sleep logs integrated with actigraphy time series: “Down” = time in bed attempting to sleep; “Up” = time between Down intervals. Analyses: Minute-by-minute activity data were exported to SPSS v24, and each participant’s data were fitted independently to a simple cosine model with 24 h periodicity. Extracted metrics included Amplitude, Acrophase, and Mesor. Daily values were also extracted to compute variability (SD across 6 days, converted to SE) to assess within-person precision and day-to-day fluctuations. Analyses primarily used continuous measures, though graphical displays grouped participants into tertiles for Amplitude and Acrophase.
Ver Hoeve et al. 2024 [33]	Mesor, Amplitude, Acrophase, R^2^	Actigraphy: Actiwatch 64, Mini-Mitter, Bend, OR, USA	Worn on non-dominant wrist	1 min	3 consecutive days at each time point (1, 4, and 16 weeks post-surgery)	Data preprocessing and wear-time validation: Sleep logs were used to document sleep–wake patterns and off-wrist periods; off-wrist periods were excluded from analyses. No further information. Analyses: Raw actigraphy data were exported to SAS and analysed using traditional cosinor modelling, simultaneously fitting 24 h and 12 h rhythms. Extracted circadian indices included Amplitude, Acrophase, Mesor, and R^2^.

Abbreviations: RAR, rest–activity rhythm; h, hours; min, minutes; s, seconds; ICC, intraclass correlation coefficient; Hz, hertz; SD, standard deviation; SE, standard error.

### 2.6. Associations Between RAR and Pain Outcomes in Different Populations

#### 2.6.1. Correlation Statistics

Two studies (Bernatchez et al. 2018 [27] and Conley et al. 2021 [28]) assessed associations between cosinor-derived parameters (Amplitude, Acrophase, Mesor, up-Mesor and down-Mesor) and pain intensity measures (worst pain in the past 24 h and abdominal pain intensity). Both studies reported correlation coefficients close to zero (ranging from −0.27 to 0.19), and none of the associations reached statistical significance (all *p* > 0.05) [27,28]. Non-parametric RAR metrics (Interdaily Stability, Intradaily Variability, and the Circadian Quotient) likewise showed non-significant correlations with pain (all *p* > 0.05) [28].

Two additional studies by Ma et al. (2014) [29] and Chang & Lin (2014) [31] evaluated indices reflecting overall rhythm strength (R24) or day–night differentiation (I > O). Higher R24 and I < O values showed statistically significant negative correlations with both worst pain and composite pain intensity (*p* ≤ 0.001) [29]. A chi-square comparison of I < O groups showed a statistically significant association between I < O classification and pain severity (*p* = 0.013) [31].

#### 2.6.2. Regression Statistics

Two studies (Mun et al. 2025 [32] and Ver Hoeve et al. 2024 [33]) used mixed-effects regression models with participant-level random intercepts to examine associations between RAR metrics and pain. In daily data, Mun et al. (2025) [32] estimated the extent to which prior-day disturbances in sleep (TST, WASO) and RARs (IV, RA) were associated with overall pain severity on the following day. In these models, Intradaily Variability was not a significant predictor (Est = −0.43, *p* = 0.375), whereas Relative Amplitude showed a statistically significant negative association with next-day pain (Est = −2.56, *p* = 0.001).

In the longitudinal dataset of Ver Hoeve et al. (2024) [33], mixed-effects linear regression models were used to examine whether individual differences in RAR measures (Mesor, Amplitude, Acrophase, R^2^), entered as time-varying predictors, were associated with differences in pain intensity and pain interference. Mesor (B = −0.20, *p* = 0.030) and Amplitude (B = −0.20, *p* = 0.026) showed significant negative associations with pain intensity, with borderline or non-significant associations for pain interference (*p* = 0.050 and *p* = 0.070, respectively). Acrophase showed a significant positive association with pain intensity (B = 0.20, *p* = 0.008) but not with pain interference (*p* = 0.145). For R^2^, mixed-effects models yielded non-significant results for both outcomes (*p* = 0.086; *p* = 0.426).

In the same longitudinal study of Ver Hoeve et al. (2024) [33], fixed-effects linear regression was used to examine whether within-person changes in RAR measures over time were associated with corresponding changes in pain intensity and pain interference. These within-person models showed statistically significant negative associations of Mesor (B = −0.51, *p* < 0.001) and Amplitude (B = −0.38, *p* = 0.001) with pain intensity and similar significant associations with pain interference (B = −0.45, *p* < 0.001; B = −0.36, *p* < 0.001). Acrophase was not significant in fixed-effects models (*p* = 0.152; *p* = 0.104). For R^2^, fixed-effects models yielded statistically significant negative associations with both pain outcomes (B = −0.19, *p* = 0.046; B = −0.19, *p* = 0.013).

#### 2.6.3. Complex Multivariate Models

Using canonical correlation analysis, Neikrug et al. (2017) [30] identified a statistically significant multivariate association (canonical r = 0.376, *p* < 0.001) between actigraphy-derived rhythm and variability measures (Amplitude, Acrophase, Mesor and their variability indices) and clinical outcomes (pain, fatigue, mood and physical impairment).

Covariance selection models showed that: (i) variability measures remained associated with the clinical outcome set after adjusting for demographics (χ^2^ = 21.23, *p* = 0.047); (ii) rhythm metrics remained associated with the clinical outcome set after adjusting for demographics and variability measures (χ^2^ = 36.24, *p* < 0.001); and (iii) rhythm metrics remained associated with the clinical outcome set beyond weekly activity averages (χ^2^ = 34.28, *p* < 0.001).

Follow-up analyses examined whether individual rhythm metrics contributed to the overall multivariate association. Using multivariate general linear models (Pillai’s trace), Amplitude, Acrophase and Mesor each showed significant multivariate associations with the clinical outcome set comprising pain, fatigue, mood and physical impairment (*p* = 0.003, 0.018, 0.007 for Amplitude, Acrophase, and Mesor, respectively). The authors reported that better pain, fatigue, mood and disability outcomes were associated with higher Amplitude and Mesor, and with lower Acrophase (earlier peak activity).

For descriptive purposes, Amplitude and Phi were grouped into three levels, and univariate contrasts were conducted for pain scores. Pain did not differ significantly across Amplitude groups (*p* = 0.229), whereas participants in the late-Phi group reported significantly higher pain compared with the early/average groups (*p* = 0.005).

The results of the correlation, regression and multivariate analyses are summarized in Table 5.

## 3. Discussion

### 3.1. Main Findings

This review is the first to provide a systematic overview of the evidence on the association between RARs and pain-related outcomes. Following a rigorous study selection process, only seven studies with predominantly low methodological quality were eligible for inclusion. Across the seven included studies, findings were heterogeneous, indicating that the association between RAR and pain depends on the type of RAR metric, the analytical approach, and the population studied.

Studies examining timing-metrics, i.e., traditional cosinor-derived metrics such as Mesor, Amplitude, and Acrophase, often reported no significant associations with pain intensity when using simple cross-sectional correlations [27,28]. In contrast, Ver Hoeve et al. (2024) demonstrated that cosinor-derived metrics become relevant when examined using longitudinal and multilevel approaches [33]. At the between-person level, higher Mesor and Amplitude, reflecting greater overall activity and stronger differentiation between the mean activity level and the peak activity, were associated with lower pain intensity, whereas a later Acrophase, indicating delayed timing of peak activity, was associated with higher pain intensity [33]. At the within-person level, postoperative increases in Mesor and Amplitude predicted reductions in both pain intensity and pain interference, reflecting concurrent changes in overall activity levels and pain outcomes over time [33]. Acrophase did not show significant within-person associations, whereas increases in R^2^, a measure of predictability and the goodness-of-fit of actigraphy-derived rhythms to a 24 h cosinor curve, were associated with modest reductions in pain [33]. Similarly, Neikrug et al. (2017) further highlighted that a combination of RAR metrics (Amplitude, Acrophase, Mesor) and their variability was significantly associated with a combined set of clinical outcomes, including pain, fatigue, mood, and physical impairment [30]. Follow-up covariance selection models demonstrated that all these metrics and their variability retained unique associations with the set of clinical outcomes after controlling for demographic factors and even after accounting for average weekly activity levels [30]. When focusing solely on pain as a clinical outcome, only Acrophase showed a clear univariate association, with later circadian timing being associated with higher pain scores, consistent with findings from Ver Hoeve et al. (2024) [30].

Metrics capturing the robustness and consolidation of the day–night rhythm, such as the Dichotomy Index (I < O) and Relative Amplitude (RA), showed associations with pain outcomes across several studies. Ma et al. (2014) and Chang & Lin (2014) reported significant negative correlations between pain intensity and I < O, indicating that individuals with a clearer separation between daytime activity and nighttime rest experience lower pain levels [29,31]. In line with this, Mun et al. (2025) found that Relative Amplitude was a significant negative predictor of daily pain severity, further supporting that a stronger contrast between active and rest periods is associated with reduced pain on a day-to-day basis [32]. Together, these studies suggest that metrics capturing rhythm robustness and consolidation may show more consistent associations with pain outcomes than traditional cosinor-derived metrics, although this pattern is based on a small number of heterogeneous studies.

Evidence regarding the regularity of rhythms was less consistent. Mun et al. (2025) reported that Interdaily Variability did not significantly predict daily pain severity within-person longitudinal analyses, and Conley et al. (2021) similarly found no significant associations between Interdaily Stability or Interdaily Variability and pain intensity [28,32]. These findings suggest that day-to-day variation or within-day fragmentation, when considered in isolation, did not yield robust associations with pain outcomes. However, other indicators of rhythm regularity may be more informative. For example, Ma et al. (2014) reported that higher R24 values, representing the autocorrelation of activity counts at a 24 h lag, calculated by correlating the activity time series of one day with that of the preceding day, were associated with lower Brief Pain Inventory scores [29]. Together with evidence from multivariate approaches (e.g., [30]), these findings suggest that rhythm regularity should be considered alongside rhythm robustness and timing rather than in isolation.

Importantly, the strength and direction of these associations appear to be population-dependent. Robustness of RARs, as captured by the Dichotomy Index (I < O), seems particularly relevant in individuals with advanced cancer [29,31], whereas Relative Amplitude was more predictive of daily pain severity in patients with temporomandibular disorder [32]. In contrast, cross-sectional analyses in palliative cancer patients [27] and adults with inflammatory bowel disease [28] found no significant associations, suggesting that study design and the timing of assessments may influence detectability. In fibromyalgia, lower Amplitude, Mesor, and later Acrophase were linked to greater pain [30], and the same thing was demonstrated within the population of post-surgical endometrial cancer patients [33]. Collectively, these findings underscore the importance of considering both population characteristics and methodological approaches when interpreting the relationship between RARs and pain. However, given the limited number of studies per population and differences in study design, these population-specific patterns should be interpreted as exploratory.

Overall, the available evidence shows that reduced robustness and consolidation of the day–night rhythm are associated with worse pain-related outcomes, whereas no such clear association is currently evident for metrics related to regularity and timing.

### 3.2. Quality and Limitations of the Reviewed Evidence

Interpretation of the findings requires careful consideration of several methodological issues.

First, the limited number of studies makes it difficult to draw generalizable conclusions. This limitation is further reinforced by the inclusion of two studies [29,31] derived from the same cohort of 68 cancer patients. These should therefore not be considered as independent sources of evidence, which further reduces the weight that can be assigned to their findings.

Second, although all studies employed wrist-worn actigraphy to assess RAR, there was substantial heterogeneity in how these measurements were conducted. Traditional approaches to monitoring behaviour in daily life, such as questionnaires, sleep logs, and diaries, are prone to recall bias and confounding by disease and lack precise timing information on these behaviours [41,42,43]. In contrast, actigraphy provides an objective, high-resolution method for capturing multiple aspects of daily behaviour, including circadian rhythms [40]. The rapid expansion of wearable technology has opened new opportunities to study these rhythms, but it also brings challenges: Actigraphy generates complex continuous movement data [40], from which multiple RAR-related metrics can be derived, each capturing a different aspect of circadian rhythmicity [40]. Additionally, studies included in this review differed across the following methodological areas:

1. Duration of monitoring: The length of actigraphy recording varied substantially across the included studies, ranging from three to fourteen days. This heterogeneity is important because the duration of monitoring directly affects the ability to capture representative sleep–wake patterns [44]. The American Academy of Sleep Medicine recommends a minimum of 72 h but notes that this may be insufficient for certain applications, such as assessing circadian rhythm disorders [45]. The SBSM Guide to Actigraphy Monitoring emphasizes that seven to fourteen days are generally preferred to provide a comprehensive picture and detect weekday–weekend variability [35]. These guidelines also acknowledge the trade-offs associated with extended monitoring, including greater participant burden, higher risk of missing data or device loss, and increased processing demands [44].
2. Sampling epoch length: The sampling epoch length varied across the included studies, either 30 s or one minute. Shorter epochs improve sensitivity and specificity for detecting sleeping and waking after sleep onset [35]. Current guidelines indicate that 30 s and 1 min epochs are the most validated and commonly used [35].
3. Non-wear detection and handling of data missingness: Approaches to identify non-wear periods and handle missing data differed across studies. Correctly distinguishing wear from non-wear is crucial, as non-wear can compromise data quality and introduce bias [35]. Ideally, participants should log off-wrist periods throughout the study [35], as done by Ver Hoeve et al. (2024). Automated algorithms have also been developed for non-wear detection [46]. For example, Mun et al. (2025) applied ActiLife’s Wear Time Validation function to identify extended inactivity indicative of non-wear [32]. Yet, studies still typically manually verify automated non-wear detection using diaries or visual inspection, indicating that the identification of wear and non-wear is not yet fully independent of human judgment. Importantly, missingness due to non-wear may not be random [46]. Participants with higher pain levels might be less consistent in wearing the device, introducing systematic bias.
4. Sleep/wake scoring: Actigraphy relies on algorithms to classify each epoch as asleep or awake. Two widely used algorithms are Cole–Kripke [38] and Sadeh [39], both validated against polysomnography and showing good overall agreement. In healthy populations, both achieve >85% agreement with polysomnography and correctly classify over 95% of sleep epochs (high sensitivity) [47]. However, wake detection is less accurate, with specificity ranging from about 60% for Cole–Kripke to 74% for Sadeh in healthy populations [47]. In patients with sleep disorders, specificity can even drop below 40% [47]. Sadeh has slightly lower sensitivity but higher specificity, making it potentially more suitable for populations with fragmented sleep, whereas Cole–Kripke may overestimate sleep in such cases [47].
5. Analytical approaches to estimate RAR metrics: There are several analytical approaches to estimate RAR metrics, including parametric, nonparametric, data-adaptive, and nonlinear dynamic methods [19]. The studies reviewed in this paper employed either parametric or nonparametric approaches. Parametric models rely on assumptions about the distribution of data and the rhythm’s shape and provide interpretable metrics such as Amplitude, Mesor, and Acrophase, but their validity depends on how well the chosen model fits the data [16]. Nonparametric methods make no assumptions about rhythm shape and are less dependent on model fit but quantify a limited set of characteristics that are not always straightforward to interpret [16]. Previous research has shown that these two approaches often yield mixed findings [19], making it difficult to compare results and to understand the relationships between circadian rhythms and health outcomes. Moreover, parametric metrics are particularly sensitive to data gaps; applying them to raw versus non-wear-cleansed data can yield substantially different results, further undermining cross-study comparability [48]. Among simple correlation and regression analyses, there was a trend toward greater significance when using non-parametric RAR metrics.

Beyond actigraphy protocols, studies also varied in design and statistical approach. Designs ranged from cross-sectional to cohort and longitudinal studies. Cross-sectional studies (e.g., [27]) typically relied on a single measurement or averaged values across days, which limits the ability to capture the impact of short-term fluctuations in RAR on pain. Longitudinal designs (Mun et al. 2014 and Ver Hoeve et al. 2024) incorporated repeated measurements within individuals, enabling intra-individual changes in RAR to be linked to changes in pain over time [32,33]. Statistical approaches also differed in how RAR metrics were operationalized. For example, Chang & Lin (2014) dichotomized participants into groups above or below a predefined rhythmicity threshold [31]. While this method simplifies interpretation, it introduces information loss and reduces statistical power.

Differences in study populations further added heterogeneity. Studies included patients with cancer at different disease stages, as well as populations with fibromyalgia and IBS, conditions that differ substantially in their underlying pain mechanisms. Within cancer populations specifically, pain mechanisms also differ by disease stage: advanced and palliative patients frequently experience mixed nociceptive and neuropathic pain, whereas survivors more commonly present with chemotherapy-induced peripheral neuropathy (CIPN), a localized peripheral mechanism [49]. Baseline pain levels may also play a role. For instance, Bernatchez et al. (2018) found no association between RAR and pain [27], which may be explained by the relatively low pain intensity in their sample compared to Ma et al. (2014), who observed stronger associations among patients with higher pain levels [29]. Within fibromyalgia patients, it has been found that a feeling of loneliness in the morning can affect the intensity of pain in the evening [50]. Psychological factors can thus contribute more to circadian pain variation than the disease itself, highlighting that even within a single diagnosis, the drivers of circadian disruption may be highly individual and linked to other factors as well. Pain is a complex and multidimensional phenomenon, and the heterogeneity across included populations suggests that disease diagnosis alone is insufficient to explain variability in RAR–pain associations. Future research should therefore characterize pain phenotype (including inflammatory, neuropathic, and central sensitization-related markers) rather than relying solely on pain severity or disease stage to understand RAR–pain relationships.

The pronounced heterogeneity across studies limits comparability and makes it difficult to identify overarching patterns, such that the reported findings are best viewed as isolated observations rather than as a coherent body of evidence.

Moreover, all included studies are observational, which calls for cautious interpretation of the findings. The observed associations do not allow for conclusions about causality or about the direction of the relationship between rest–activity rhythms and pain. Bidirectional relationships, as well as the influence of unmeasured third factors such as mood, activity levels, or medication use, therefore remain plausible.

Finally, risk-of-bias assessment reflected generally low methodological quality: one study raised some concerns, four were high risk, and two were very high risk. The three main limitations across studies were insufficient control for confounders, short actigraphy monitoring periods in most of the studies, and selective exclusion of participants from the analysis or missing actigraphy data, all of which may compromise the reliability and generalizability of the observed RAR–pain associations.

### 3.3. Circadian Rhythms and Pain

The findings of our review may fit within a broader body of literature showing that sleep characteristics, physical activity levels, and other behavioural factors are related to pain [51,52,53]. Although these bidirectional associations help explain why behavioural factors influence pain, they do not explain how their timing and regularity shape pain experiences. In this regard, circadian rhythms have recently gained attention as a novel and underexplored contributor to pain [54].

Increasing evidence indicates that pain itself is under circadian control [54,55,56] and that pain modulation pathways and circadian regulation interact via different mechanisms [57]: At the circuit level, the suprachiasmatic nucleus (SCN) regulates pain processing through a multisynaptic pathway involving the subparaventricular zone (SPZ) and the lateral parabrachial nucleus (LPB), forming a bidirectional network in which nociceptive and circadian signals reinforce each other. At the molecular level, clock genes (e.g., BMAL1, CLOCK, PER, CRY) drive rhythmic expression of nociceptive mediators (including substance P, NMDA receptors, and cytokines), while inflammatory signals reciprocally disrupt these clocks. Glial cells possess intrinsic circadian rhythms and critically mediate neuroinflammation and SCN desynchronization. Importantly, different pain conditions (e.g., inflammatory vs. neuropathic) disrupt these systems in distinct ways, and circadian fluctuations in hormones and neurotransmitters further modulate pain and treatment response [57].

Previous research has mainly focused on describing daily pain fluctuations or the effects of circadian disruption [57,58,59,60], offering limited insight into whether the organization and robustness of everyday activity patterns themselves relate to pain. Our systematic review addresses this gap by synthesizing evidence on RAR metrics and pain outcomes, positioning RAR as a potentially modifiable pathway linking circadian regulation to pain.

The present findings suggest that the timing and consolidation of activity and rest may be more relevant to pain than overall activity levels. Metrics such as the Dichotomy Index (I < O) and Relative Amplitude capture how well periods of activity and rest are segregated across the 24 h cycle, and these metrics showed the strongest associations with pain. Individuals with more pronounced and stable day–night activity patterns tend to report lower pain levels, indicating that clearly defined behavioural rhythms support circadian alignment across systems involved in pain processing.

However, patients with chronic pain frequently exhibit poorly consolidated daily activity patterns, characterized by periods of overactivity, during which patients attempt to complete tasks or “push through” pain, followed by prolonged periods of inactivity or bed rest during pain flares [61,62,63]. Overall, they spend more time lying or sitting than healthy individuals [64], frequently accompanied by pain-related sleep disturbances and nocturnal wakefulness [65]. Together, these patterns blur the distinction between day and night and undermine circadian signalling.

In addition to rhythm robustness, the timing of peak activity may also hold clinical relevance for pain. A later Acrophase, reflecting a shift of peak activity toward the evening, corresponds to evening chronotypes, who often exhibit greater pain sensitivity than morning chronotypes [66,67]. Several mechanisms may explain this association, which has already been shown in the literature. First, affective functioning is central to pain perception: negative mood states enhance pain sensitivity, and evening types often report less favourable mood profiles and higher levels of depressive symptoms than morning types [68]. Second, circadian regulation of cortisol may contribute: evening types tend to have lower daytime cortisol levels and attenuated cortisol awakening responses, both associated with increased pain sensitivity [68]. Third, sleep patterns and social jetlag exacerbate pain in individuals with later Acrophase, as delayed sleep–wake timing can lead to sleep deprivation in social situations [68]. Together, these factors provide a plausible explanation for the link between late Acrophase and elevated pain observed across multiple studies in this systematic review, but more research about the timing of physical activity and its effects on pain and sleep is needed.

Finally, regular and well-timed physical activity is a potent zeitgeber that strengthens the day–night rhythm, whereas physical inactivity and irregular activity patterns are increasingly recognized as risk factors for pain [11,52,53].

RARs have also been studied in relation to conditions beyond pain. In mood disorders, irregular and delayed RAR profiles are linked to higher levels of depressive and manic–hypomanic symptoms [69,70]. In dementia research, greater rhythm fragmentation relates to neurodegenerative biomarkers, lower amplitude predicts future cognitive decline, and reduced 24 h rhythm fit is associated with executive dysfunction [24]. Similarly, in cardiometabolic research, more stable and less fragmented rhythms are associated with lower odds of cardiovascular disease, hypertension, and obesity, even after adjustment for traditional lifestyle factors [71].

Although further longitudinal research is needed, this suggests that circadian disorganization may represent a transdiagnostic vulnerability factor rather than a pain-specific mechanism. From this broader perspective, pain may constitute one manifestation of a more generalized circadian dysregulation phenotype, affecting multiple interconnected physiological systems.

### 3.4. Strengths and Limitations of This Review and Protocol Deviation

This review followed rigorous methodological standards. The protocol was prospectively registered to enhance transparency and reduce the risk of bias. A comprehensive search was conducted across multiple databases, and study screening and data extraction were performed independently by two reviewers. Similarly, risk of bias was assessed independently by two reviewers using a standardized instrument, ensuring consistent evaluation of study quality.

Despite these strengths, the review has several limitations. First, a meta-analysis could not be performed due to substantial heterogeneity in RAR measurement approaches, study designs, and pain populations. Second, despite an extensive search, the possibility of publication bias cannot be excluded, as unpublished or non-English studies may have been missed. Third, the included studies predominantly examined Western pain populations; therefore, the findings may not be generalizable to non-Western populations.

A protocol deviation occurred during the review process. The initial protocol aimed to capture all of the literature examining circadian regulation in relation to pain, including chronotype, RAR, and circadian dysregulation. During screening, it became apparent that these domains were conceptually and methodologically distinct, making combined synthesis impractical. Therefore, the scope of this review was narrowed to focus specifically on RAR, ensuring a coherent and interpretable synthesis while recognizing that the other domains warrant separate analyses.

### 3.5. Future Research Recommendations and Clinical Implications

While circadian principles are already proposed to some extent in phototherapeutic [72,73] and pharmacological interventions [74,75], future research is needed to clarify which specific RAR metrics are most strongly associated with pain outcomes and to understand the mechanisms underlying these associations, exploring why certain features of circadian rhythmicity relate to pain perception and severity. A deeper mechanistic understanding is essential before translating these findings into clinical practice, designing interventions, or testing targeted strategies to modulate RAR for pain management. Firstly, standardized measurement approaches are needed. Actigraphy offers a powerful tool for assessing RAR in large chronic pain populations, yet current practices for sampling, data processing, and analysis vary widely, limiting comparability. An urgent consensus on minimum data quality standards—including thresholds for non-wear detection and sampling density—is needed to enable valid metric comparisons and reproducible research. While general guidelines and flowcharts exist to support actigraphy use [35], dedicated guidance specifically for RAR measurement in different pain populations and with regard to different pain mechanisms would greatly improve data quality, enable more reliable cross-study comparisons, and facilitate the translation of research findings into clinical practice.

Despite these gaps, the results of this review suggest cautious yet promising clinical considerations for pain rehabilitation. Lifestyle interventions that focus on the timing and regularity of sleep and physical activity could provide low-risk, non-invasive strategies to improve pain outcomes [60]. Educating patients about the importance of maintaining a clear and well-consolidated day–night rhythm, characterized by regular daytime activity and protected nighttime rest, may offer a simple yet effective preventive approach. Early evidence already suggests that integrating simple circadian-alignment practices into daily routines may support sustainable improvements in sleep regularity and health outcomes [75]. Future research should further investigate how chronobiologically-informed interventions can optimize pain management in clinical populations.

In this context, the rapid advancement of wearable devices could enable remote monitoring of rest–activity rhythms to assess and support the behavioural separation between activity and rest as a feasible component of routine care. While further research is needed to confirm and refine these strategies, they already point toward practical avenues to support patients with chronic pain.

## 4. Materials and Methods

### 4.1. Systematic Review Protocol

This study follows the Preferred Reporting Items for Systematic Reviews and Meta-Analyses (PRISMA) guidelines [76]. The PRISMA checklist is included in the Supplementary Materials. The protocol of this review was preregistered on PROSPERO (CRD420251052792).

### 4.2. Eligibility Criteria

This systematic review includes observational studies across healthy and clinical populations that objectively quantify RAR over multiple days (at least 3 days) and report statistical associations with pain outcomes (e.g., pain prevalence, sensitivity, intensity, duration, or interference). Table 6 outlines the inclusion and exclusion criteria that guided the selection of studies for this review.

### 4.3. Search Strategy and Study Selection Process

Systematic searches were conducted in PubMed (MEDLINE), Embase, and Web of Science Core Collection, with the final search performed on 20 May 2025. The full search strategy and search strings are provided in Appendix A. Search terms were structured around three core concepts: (1) rest–activity rhythm or circadian activity patterns, (2) actigraphy or accelerometry, and (3) pain and pain-related outcomes. Both controlled vocabulary (e.g., MeSH and Emtree terms) and free-text keywords were combined using Boolean operators (AND, OR).

All search results were exported to EndNote (Clarivate Analytics) for reference management and subsequently imported into Rayyan (Qatar Computing Research Institute) to facilitate systematic screening and documentation of decisions. After duplicate removal, two reviewers (M.D.d. and A.V.S.) independently screened all titles and abstracts. Discrepancies or uncertain cases were resolved through consensus before proceeding to full-text screening, which was again performed independently by both reviewers. Finally, reference lists of eligible studies and relevant reviews were screened manually to identify additional publications not captured through electronic searches.

### 4.4. Data Extraction

Data from all included studies were extracted systematically using a predefined data extraction form. Data extraction was done by A.V.S. and checked by M.D.d. and consisted of the following items:

- Study identification: first author, year of publication, country, and study design.
- Population characteristics: sample size, demographic information (age, sex), health or pain condition, inclusion/exclusion criteria, study setting and how recruitment was carried out.
- RAR assessment details: device type, recording duration, software or algorithm used for data preprocessing.
- Specific RAR metrics reported (e.g., Interdaily Stability, Intradaily Variability, Relative Amplitude, Acrophase, Amplitude, transition probability).
- Pain-assessment details: the assessment method used, the timing or time points of assessment, and the measurement procedure.
- Specific pain-related outcomes (e.g., pain intensity, sensitivity, threshold, frequency, interference, or duration).
- Statistical analyses and effect estimates: reported associations between RAR metrics and pain outcomes, including correlation coefficients, regression estimates, or other summary statistics.
- Confounders and covariates: variables controlled for in the analysis.
- Risk of bias assessment outcomes (as evaluated using the ROBINS-E tool; see Section 2.5).

### 4.5. Risk of Bias Assessment

The risk of bias for all included studies was assessed using the ROBINS-E (Risk Of Bias In Non-randomized Studies of Exposures) tool, which provides a structured framework for evaluating potential biases in observational studies examining exposure–outcome relationships [77].

Two reviewers (M.D.d. and A.V.S.) independently assessed each study across the seven ROBINS-E domains: (1) bias due to confounding factors, (2) bias arising from measurement of exposure, (3) bias in selection of participants for the study or the analysis, (4) bias due to post-exposure interventions, (5) bias due to missing data, (6) bias arising from measurement of outcomes, and (7) bias in selection of the reported result [77].

To ensure consistency and reproducibility, the two reviewers established several predefined criteria prior to the risk of bias assessment. Relevant confounders were identified through a review of the literature to capture variables known to correlate with both RAR and pain but unlikely to fully account for their association (i.e., mediators). These confounders included psychological distress (primarily depression) [78], sociodemographic factors (age, sex, ethnicity, socioeconomic status, income) [79], BMI [80], type of work (e.g., shift work) [81,82], and cardiovascular or metabolic comorbidities (e.g., diabetes) [71]. During the risk of bias assessment, studies were evaluated on whether and how they accounted for these predefined confounders. This information was incorporated into the scoring of the relevant risk of bias items, with higher scores assigned to studies that adequately adjusted for key confounding variables.

Second, a minimum standard exposure window for valid RAR assessment was established to ensure construct validity. Based on the prior methodological literature [38] and expert consensus within the review team, studies were required to include at least seven consecutive valid days of actigraphy recording to be considered sufficiently representative.

Each domain was then judged as having a *low risk of bias, some concerns, high risk of bias, or very high risk of bias*, following the ROBINS-E guidance and algorithm. Any discrepancies between reviewers (M.D.d. and A.V.S.) were discussed and resolved through consensus, and if required, there was the option to involve a third researcher (L.D.B.). To quantify inter-rater agreement prior to consensus, Cohen’s kappa (κ) was calculated across all domains.

## 5. Conclusions

This review highlights preliminary evidence that more robust day–night activity rhythms may be linked to lower pain outcomes. Findings are limited by methodological heterogeneity and small study numbers, emphasizing the need for standardized assessment and mechanistic research. Advancing this field could inform personalized, rhythm-based strategies for pain management.

## Figures and Tables

**Table 1:** Study characteristics of the included studies.

Study	Country	Study Design	Population	Total Sample Size (N)	Age	Female (%)	Study Setting & Recruitment	In- & Exclusion Criteria
Bernatchez et al. 2018 [27]	Canada	Cross-sectional observational study with prospective data collection	Palliative cancer patients	57	Mean (SD) = 65.9 (10.4)	47.3	The study was conducted among community-dwelling advanced cancer patients receiving palliative care in Quebec City, Canada, recruited from an outpatient palliative care clinic and a day care centre of a palliative care hospice.	✓ Diagnosis of advanced cancer ✓ ECOG performance status of 2 or 3 ✓ Age ≥ 18 years ✓ Able to read and understand French ✓ Able and willing to provide informed consent ✓ Live within 90 min of L’Hôtel-Dieu de Québec ✗ Current delirium, dementia, or severe cognitive impairment ✗ MMSE score ≤ 23 ✗ Suicidal thoughts (score ≥ 1 on items 4 or 5 of SSI) ✗ Suicide attempt in past 5 years
Chang & Lin 2014 [31]	Taiwan	Observational, prospective cohort study	Cancer patients experiencing pain	68	Median (25–75% IQR) = 54.0 (43.3–61.0)	50.0	Clinical: Oncology ward of a teaching hospital in Taiwan—Participants were hospitalized for cancer treatment or symptom control and recruited between August 2006 and October 2007.	✗ Surgical treatment within previous 3 months ✗ Bedridden ✗ Unable to wear wrist actigraphy
Conley et al. 2021 [28]	U.S.	Cross-sectional observational study with prospective data collection	Patients with IBD	37	Mean (SD) = 38 (13.8)	56.8	Single GI clinic at an academic medical centre in the Northeastern United States. Participants were approached after their IBD clinic visit and provided informed consent.	✓ Age 18–60 years ✓ Diagnosed with IBD ✗ Blindness ✗ Unable to read/write English ✗ No internet access ✗ Pregnancy or breastfeeding ✗ Night or rotating shift work ✗ Recent travel across ≥2 time zones ✗ Surgery in past 4 weeks ✗ Use of sleep medications ✗ Severe psychiatric or neurological conditions ✗ Data collection in week after daylight saving time change
Ma et al. 2014 [29]	Taiwan	Cross-sectional observational study with prospective data collection	Advanced cancer patients	68	Mean (SD) = 52.40 (11.87)	50.0	Clinical: Oncology inpatient departments of a teaching hospital in northern Taiwan—Participants were recruited from oncology inpatient units of a medical centre in the Taipei area.	✓ Diagnosed with advanced cancer ✓ Currently experiencing cancer pain ✓ Age ≥ 18 years ✓ No mobility limitations ✓ No surgery in past 3 months ✓ Able to communicate in Mandarin or Taiwanese dialect
Mun et al. 2025 [32]	U.S.	Longitudinal observational study	Patients with TMD and insomnia symptoms	140	Mean (SD) = 37 (11.4)	100		✓ Female, aged 18–60 years ✓ Positive TMD diagnosis (diagnostic TMD criteria) ✓ Myalgic or arthralgic facial pain ≥3 months ✓ Facial pain ≥10 days in past 30 days ✓ Average pain severity ≥ 3 (0–10 scale) ✓ ISI > 8 and PCS > 8 ✓ Sleep initiation/maintenance problems >3/week for ≥1 month ✓ Stable non-opioid pain medication use for past 30 days ✓ Willingness for 4-week washout if using opioids/benzodiazepines/tricyclic antidepressants ✓ Willingness to use contraception (if applicable) ✓ Postmenopausal ≥12 months (if applicable) ✓ Ability and willingness to comply with study procedures ✗ BMI > 35 ✗ Resting BP > 140/90 mmHg ✗ TMJ surgery, neoplasm, or injury in past 6 months ✗ Scheduled TMJ surgery during study ✗ Medical conditions affecting sleep/CNS/peripheral nerves ✗ Raynaud’s syndrome ✗ Unstable severe psychiatric disorder ✗ Problematic substance/alcohol use in past 6 months ✗ Use of opioids/benzodiazepines/tricyclic antidepressants ✗ Night shift work or significant sleep variability ✗ CES-D ≥ 27 or suicidal ideation ✗ Positive urine test for drugs/alcohol ✗ Positive pregnancy test ✗ RDI > 15 or PLMI > 15 (based on PSG) ✗ Any other factor interfering with study completion
Neikrug et al. 2017 [30]	U.S.	Cross-sectional observational study with prospective data collection	FMS patients	292	Mean (SD) = 45.1 (11.1)	93.2	Home-based. Recruitment via flyers, radio ads, mailings, and referrals from local dental professionals; data collection occurred in participants’ homes.	✓ Diagnosed with FMS ✗ Missing/incomplete actigraphy (>1 night or >8 h a day) ✗ Reversed diurnal rhythm (peak activity 11 PM–8 AM)
Ver Hoeve et al. 2024 [33]	U.S.	Longitudinal observational study	Endometrial cancer survivors	69	<64 years: 68.1%/≥65 years: 31.9%	100	Research centre + home-based—recruitment via referrals from a University Pain Centre, community physicians, and advertisements.	✓ Age ≥ 18 years ✓ Scheduled for primary surgery for endometrial malignancy

*✓* = inclusion criterion*; ✗ =* exclusion criterion. Abbreviations: ECOG, Eastern Cooperative Oncology Group; MMSE, Mini-Mental State Examination; GI, gastrointestinal; IBD, inflammatory bowel disease; TMD, temporomandibular disorder; ISI, Insomnia Severity Index; PCS, Pain Catastrophizing Scale; BMI, Body Mass Index; BP, blood pressure; TMJ, Temporomandibular Joint; CES-D, Centre for Epidemiologic Studies Depression Scale; RDI, Respiratory Disturbance Index; PLMI, Periodic Limb Movement Index; PSG, polysomnography; FMS, Fibromyalgia Syndrome.

**Table 2:** Pain instrument characteristics.

Study	Pain Construct	Timeframe Questions	Instrument	Number of Items	Scale	Assessment Time Points	Scoring Approach
Bernatchez et al. 2018 [27]	Pain intensity: worst pain	“Worst pain during the previous 24 h”	Pain diary	Single BPI-derived item	0–10 NRS	Daily for 7 consecutive days	Average of the 7 daily worst pain ratings
Chang & Lin 2014 [31]	Pain severity	NR	BPI—Chinese version	NR	0–10 NRS per item	Single time point (baseline, before actigraphy)	Mild (1–4), moderate (5–6), severe (7–10)
Conley et al. 2021 [28]	Abdominal pain	Over the previous 7 days	PROMIS-GI Symptom Scales—symptom: belly pain	NR	NR	Single time point (baseline, before actigraphy)	Raw score converted to T-score (M = 50, SD = 10); higher = more severe pain
Ma et al. 2014 [29]	Pain intensity & interference	Pain intensity items: “Worst pain in the last 24 h”/“Least pain in the last 24 h”/“Average pain”/“Pain right now” Interference items: “During the past 24 h”	BPI—Chinese version	11 (4 on pain intensity, 7 on pain interference)	0–10 NRS per item	Single time point (baseline, before actigraphy)	Intensity: separate items (worst, least, average, current). Interference: mean of 7 items.
Mun et al. 2025 [32]	Pain severity: average pain	“Average pain experienced throughout the entire day”	Evening daily diary (IVR system)	1	0–10 NRS	Daily for 14 consecutive days	Average score per day
Neikrug et al. 2017 [30]	Pain severity	“How pain interferes with different life activities and how severe it was over the last week”	MPI—pain severity subscale	NR	NR	Single time point (baseline, before actigraphy)	Mean score on the pain severity subscale
Ver Hoeve et al. 2024 [33]	Pain intensity & interference	NR	BPI—short form	NR	0–10 NRS	1, 4, and 16 weeks post-surgery	Composite scores for pain intensity & interference separately—clinical cut-off ≥5

Abbreviations: BPI, Brief Pain Inventory; NRS, Numeric Rating Scale; PROMIS, Patient-Reported Outcomes Measurement Information System; GI, gastrointestinal; M, mean; SD, standard deviation; IVR, Interactive Voice Response; MPI, Multidimensional Pain Inventory; NR, not reported.

**Table 3:** Overview of rest–activity rhythm (RAR) metrics, categorized by analytical approach, including metric name and definition. Sources: [19,20,21].

Parameter Type	Parameter	Definition	Reported in Study
Parametric	Mesor (M)	The Midline Estimating Statistic of Rhythm (Mesor) represents the mean level of activity within a 24 h circadian pattern. Conceptually, it corresponds to the baseline around which the cosinor curve oscillates and is equivalent to the estimated constant term in the cosinor regression model.	[27,28,30,33]
	Amplitude (A)	Amplitude quantifies the extent of predictable change within a circadian cycle. It is defined as the difference between the peak of the rhythm and the Mesor, reflecting the strength of the oscillation.	[27,28,30,33]
	Acrophase/Phi (φ)	Acrophase indicates the timing of the peak of the rhythm within a cycle. It represents the time at which the highest values recur, typically expressed in degrees relative to a reference time set at 0°, with 360° corresponding to one complete cycle.	[27,28,30,33]
	R^2^	The coefficient of determination (R^2^) reflects the goodness-of-fit of the cosinor model to the observed rest–activity data. A high R^2^ indicates that the circadian pattern is well captured by the cosinor curve, whereas a low R^2^ suggests irregular or flattened rhythms. R^2^ serves as a quantitative measure of the fit quality.	[27,33]
	Circadian Quotient (CQ)	Circadian Quotient reflects the ratio of the Amplitude to Mesor. Higher CQ values indicate a more robust and well-defined day–night activity pattern, whereas lower values reflect a dampened or flattened rhythm.	[28]
Non-parametric	Interdaily Stability (IS)	Interdaily Stability quantifies the consistency of a 24 h rhythm across multiple days. High IS values indicate a stable and reproducible daily activity pattern, whereas low IS reflects irregularity in the day-to-day rhythm.	[28]
	Intradaily Variability (IV)	Intradaily Variability measures the fragmentation of the rhythm within a day. High IV values indicate frequent and abrupt transitions between activity and rest, reflecting a fragmented circadian pattern, whereas low IV represents a smooth, consolidated rhythm.	[28,32]
	Relative Amplitude (RA)	Relative Amplitude represents the difference between the most active period and the least active period within a 24 h cycle. High RA values indicate a pronounced distinction between active and rest periods, reflecting a robust circadian rhythm.	[32]
	Dichotomy Index (I < O)	The Dichotomy Index compares activity levels during in-bed versus out-of-bed periods. High values indicate a clear distinction between sleep and wake states, reflecting preserved rest–activity segregation, whereas low values indicate poor differentiation between sleeping and waking periods.	[29,31]
	Autocorrelation Coefficient (R24)	R24 represents the autocorrelation of activity counts at a 24 h lag. Calculated by correlating the activity time series with itself shifted by 24 h. High R24 values indicate a regular, robust circadian rhythm, while low values reflect irregular or flattened rhythms.	[29]

**Table 5:** Overview of the results of the correlation, regression and multivariate analyses per included study.

Study	RAR Metric	M ± SD/(Range)/[IQR]	Pain Measure	M ± SD/(Range)	Statistical Analysis	Reported Statistic	Significance
Bernatchez et al. 2018 [27]	Amplitude	47.0 (5.4–178.8)	Worst pain (24 h)	3.9	Spearman correlation	ρ = 0.06	ns (*p* > 0.05)
	Acrophase	13:35 (10:34–20:10)				ρ = 0.08	ns (*p* > 0.05)
	Mesor	45.4 (3.6–167.8)				ρ = 0.18	ns (*p* > 0.05)
	Up-Mesor	8:18 (2:00–14:00)				ρ = −0.14	ns (*p* > 0.05)
	Down-Mesor	19:23 (16:20–22:00)				ρ = 0.03	ns (*p* > 0.05)
	R^2^	0.27 (0.09–0.51)				ρ = −0.07	ns (*p* > 0.05)
Conley et al. 2021 [28]	Amplitude	66.95 ± 21.11	Abdominal pain	57.38 ± 12.60	Pearson correlation	r = 0.185	ns (*p* > 0.05)
	Acrophase	15:10 ± 1.47				r = 0.167	ns (*p* > 0.05)
	Mesor	85.42 ± 22.88				r = −0.030	ns (*p* > 0.05)
	Circadian Quotient	0.79 ± 0.13				r = −0.034	ns (*p* > 0.05)
	Interdaily Stability	0.52 ± 0.11				r = −0.274	ns (*p* > 0.05)
	Intradaily Variability	0.84 ± 0.21				r = 0.054	ns (*p* > 0.05)
Ma et al. 2014 [29]	R24	0.19 ± 0.16	Worst pain	5.47 ± 2.70	Pearson correlation	r = −0.51	*p* < 0.001
			Composite pain intensity	3.18 ± 2.15		r = −0.47	*p* < 0.001
	I > O	85.29 ± 0.13	Worst pain			r = −0.42	*p* < 0.001
			Composite pain intensity			r = −0.41	*p* = 0.001
Chang & Lin (2014) [31]	I > O group: (1) I < O ≥ 89.5% (2) I < O ≤ 89.4%		Pain severity: (1) mild (1–4) (2) moderate (5–6) (3) severe (7–10)	Regular I < O ≥ 89.5%: 55.9% mild, 17.6% moderate, 26.5% severe. Disrupted I < O ≤ 89.4%: 23.5% mild, 29.4% moderate, 47.1% severe.	Chi-squared test	NR	*p* = 0.013
Mun et al. (2025) [32]	Intradaily Variability	0.54 ± 0.12 [0.47, 0.60]	Daily pain severity		Linear mixed effects regression: Sleep and RAR measures entered as fixed-effects; daily pain severity as the outcome.	Est = −0.43, SE = 0.49, 95% CI [−1.38, 0.52]	*p* = 0.375
	Relative Amplitude	0.92 ± 0.09 [0.91, 0.97]				Est = −2.56, SE = 0.75, 95% CI [−4.03, −1.09]	*p* = 0.001
Ver Hoeve et al. (2024) [33]	Mesor		Pain intensity	1 week post-surgery: 3.8 ± 1.9 4 weeks post-surgery: 2.1 ± 2.0 16 weeks post-surgery: 2.1 ± 2.3	Mixed-effects regression model: RAR measures entered as time-varying predictors; outcome: pain intensity or pain interference; adjusted for time since surgery, age, BMI, disease stage, surgery type, and adjuvant therapy.	B = −0.20	*p* = 0.030
					Fixed-effects regression model: examining time-varying changes in RAR measures as predictors of pain intensity or pain interference; models adjusted for time since surgery.	B = −0.51	*p* < 0.001
			Pain interference	1 week post-surgery: 4.3 ± 2.6 4 weeks post-surgery: 2.1 ± 2.4 16 weeks post-surgery: 2.0 ± 2.6	Mixed-effects regression model	B = −0.14	*p* = 0.050
					Fixed-effects regression model	B = −0.45	*p* < 0.001
	Amplitude		Pain intensity		Mixed-effects regression model	B = −0.20	*p* = 0.026
					Fixed-effects regression model	B = −0.38	*p* = 0.001
			Pain interference		Mixed-effects regression model	B = −0.12	*p* = 0.070
					Fixed-effects regression model	B = −0.36	*p* < 0.001
	Acrophase		Pain intensity		Mixed-effects regression model	B = 0.20	*p* = 0.008
					Fixed-effects regression model	B = 0.18	*p* = 0.152
			Pain interference		Mixed-effects regression model	B = 0.09	*p* = 0.145
					Fixed-effects regression model	B = 0.16	*p* = 0.104
	R^2^		Pain intensity		Mixed-effects regression model	B = −0.13	*p* = 0.086
					Fixed-effects regression model	B = −0.19	*p* = 0.046
			Pain interference		Mixed-effects regression model	B = −0.05	*p* = 0.426
					Fixed-effects regression model	B = −0.19	*p* = 0.013
Neikrug et al. (2017) [30]	Set XR: Amplitude, Acrophase, Mesor + Set XSE: Amplitude variability, Acrophase variability, Mesor variability		Clinical outcomes (set Y): pain, fatigue, mood, and physical impairment		Canonical correlation between actigraphy-derived measures (set XR + XSE) and clinical outcomes (set Y)	r = 0.376, R^2^ = 0.14, Wilks’ Lambda = 0.799	*p* < 0.001
					Covariance selection models (likelihood ratio tests): i: Y ⟂ XSE|D ii: Y ⟂ XR∣D, XSE iii: Y ⟂ XR∣weekly activity average	(i) Y ↔ XSE|D: χ^2^(12) = 21.23 (ii) Y ↔ XR|D + XSE: χ^2^(12) = 36.24 (iii) Y ↔ XR|weeklyavg: χ^2^(12) = 34.28	(i) *p* = 0.047 (ii) *p* < 0.001 (iii) *p* < 0.001
	Amplitude		Clinical outcomes (set Y): pain, fatigue, mood, and physical impairment		Multivariate general linear models (Pillai’s trace)	NR	*p* = 0.003
	Acrophase					NR	*p* = 0.018
	Mesor					NR	*p* = 0.007
	Amplitude: Low/Avg/High		Pain	- Low: 4.1 - Avg: 3.9 - High: 4.0	Univariate ANOVAs with planned (a priori) contrasts	NR	Low vs. Avg/High *p* = 0.229
	Acrophase: Early/Avg/Late			- Early 3.9 - Avg 3.9 - Late 4.2		NR	Late vs. Early/Avg: *p* = 0.005

↔ indicates a bidirectional or non-directional association; ⟂ indicates independence between variables; | indicates conditioning on or adjustment for a variable. Abbreviations: RAR, rest–activity rhythm; ns, not significant; NR, not reported; h, hours; avg, average.

**Table 6:** Inclusion and exclusion criteria used to determine study eligibility.

	Inclusion	Exclusion
Study design	Observational studies (cross-sectional, case–control, longitudinal, or cohort) reporting associations between objectively measured RAR metrics and pain-related outcomes.	Interventional clinical trials not reporting associations, case reports, qualitative studies, reviews, editorials, and conference abstracts without full data.
Population	Adults and/or children from the general population or clinical populations with acute or chronic pain conditions.	Animal studies; studies focusing solely on people with sleep disorders without pain-related outcomes.
Exposure	Studies that quantify RAR using actigraphy or similar accelerometer-based instruments, reporting at least one of the following metrics: Amplitude, Acrophase, Interdaily Stability (IS), Intradaily Variability (IV), Relative Amplitude (RA) or other validated RAR metrics.	Studies that only report total activity counts, sleep duration, or circadian preference (e.g., chronotype) without a specific RAR metric.
Outcome	Studies reporting pain-related outcomes, including but not limited to: pain intensity or severity, pain sensitivity or pain pressure thresholds, pain frequency or pain duration, pain interference or impact on functioning.	Studies reporting only non-pain outcomes (e.g., fatigue, mood).
Language	Peer-reviewed articles published in English.	Languages other than English.
Publication criteria	No restriction on publication year, only included if we have access to the full text.	No full text available.

## Data Availability

The original contributions presented in this study are included in the article. Further inquiries can be directed to the corresponding author.

## Supplementary Materials

The following supporting information can be downloaded at: https://www.mdpi.com/article/10.3390/clockssleep8020032/s1, Table S1: PRISMA 2020 Checklist [76].

## Abbreviations

The following abbreviations are used in this manuscript:

RAR	Rest–Activity Rhythm
BPI	Brief Pain Inventory
NRS	Numeric Rating Scale
PROMIS	Patient-Reported Outcomes Measurement Information System
MPI	Multidimensional Pain Inventory
M	Mesor
A	Amplitude
φ	Phi or Acrophase
CQ	Circadian Quotient
IS	Interdaily Stability
IV	Intradaily Variability
RA	Relative Amplitude
I > O	Dichotomy Index

## Appendix A

PUBMED

(((“circadian rhythm”[MeSH Terms] OR Chronotype [Title/Abstract] OR “diurnal type”[Title/Abstract] OR “evening type”[Title/Abstract] OR “morning type”[Title/Abstract] OR “intermediate type”[Title/Abstract] OR “sleep–wake cycle”[Title/Abstract] OR chronobiology [Title/Abstract])) AND ((“pain”[MeSH Terms] OR pain [Title/Abstract] OR “acute pain”[Title/Abstract] OR “chronic pain”[Title/Abstract] OR nociception[MeSH Terms] OR nocicept*[Title/Abstract])))

EMBASE

((‘circadian rhythm’/exp OR chronotype:ti,ab OR “diurnal type”:ti,ab OR “evening type”:ti,ab OR “morning type”:ti,ab OR “intermediate type”:ti,ab OR “sleep–wake cycle”:ti,ab OR chronobiology:ti,ab) AND (“pain”/exp OR pain:ti,ab OR “acute pain”:ti,ab OR “chronic pain”:ti,ab OR “nociception”/exp OR nocicept*:ti,ab))

WEB OF SCIENCE

TS = ((“circadian rhythm” OR chronotype OR “diurnal type” OR “evening type” OR “morning type” OR “intermediate type” OR “sleep–wake cycle” OR chronobiology) AND (pain OR “acute pain” OR “chronic pain” OR nociception OR nocicept*))

SCOPUS

TITLE-ABS-KEY(“circadian rhythm” OR chronotype OR “diurnal type” OR “evening type” OR “morning type” OR “intermediate type” OR “sleep–wake cycle” OR chronobiology AND (pain OR “acute pain” OR “chronic pain” OR nociception OR nocicept*))
